# Body Roundness Index Is Better Correlated with Insulin Sensitivity than Body Shape Index in Young and Middle-Aged Japanese Persons

**DOI:** 10.1089/met.2023.0175

**Published:** 2024-03-14

**Authors:** Norimitsu Murai, Naoko Saito, Rie Oka, Sayuri Nii, Hiroto Nishikawa, Asami Suzuki, Eriko Kodama, Tatsuya Iida, Kentaro Mikura, Hideyuki Imai, Mai Hashizume, Rie Tadokoro, Chiho Sugisawa, Toru Iizaka, Fumiko Otsuka, Shun Ishibashi, Shoichiro Nagasaka

**Affiliations:** ^1^ Division of Diabetes, Metabolism and Endocrinology, Showa University Fujigaoka Hospital, Yokohama, Japan.; ^2^ Division of Endocrinology and Metabolism, Department of Medicine, Jichi Medical University, Tochigi, Japan.; ^3^ Department of Internal Medicine, Hokuriku Central Hospital, Toyama, Japan.

**Keywords:** oral glucose tolerance test, insulin sensitivity, body shape index, body roundness index, waist circumference

## Abstract

**Aims:**

The present study aimed to clarify the relationships between novel and traditional anthropometric indices and insulin sensitivity (SI) in young and middle-aged Japanese persons with normal glucose tolerance (NGT), and middle-aged Japanese persons with NGT and glucose intolerance.

**Methods:**

Plasma glucose and insulin levels were measured in 1270 young (age <40 years) and 2153 middle-aged persons with NGT (*n* = 1531) and glucose intolerance (*n* = 622) during a 75-g oral glucose tolerance test. Height (Ht), weight, and waist circumference (WC) were measured. The body mass index (BMI), WC, and the WC/Ht ratio were used as traditional anthropometric indices. A body shape index (ABSI) and the body roundness index (BRI) were calculated as novel indices. Indices of SI (Matsuda index and 1/homeostasis model assessment of insulin resistance) were calculated and compared with anthropometric indices.

**Results:**

The ABSI showed a weak correlation with SI indices in all groups. The BRI showed almost the same correlation with SI indices as the BMI, WC, and WC/Ht in all groups. The inverse correlation between each of the anthropometric indices other than ABSI and SI indices was weak in young persons, at 0.16–0.27 (Spearman's ρ values), but strong in middle-aged persons, at 0.38–1.00. On receiver-operating characteristic (ROC) curve analysis for detection of insulin resistance, the ABSI had a lower area under the ROC curve (AUC) than the other anthropometric indices, and the BRI and the WC/Ht ratio showed similar AUCs. The AUCs for the BRI and WC/Ht ratio were the highest in middle-aged men with NGT and glucose intolerance.

**Conclusions:**

The BRI, not the ABSI, was better correlated with SI in young and middle-aged Japanese persons. The BRI and WC/Ht ratio were comparable in their correlations with SI and the detection of insulin resistance in the participants of the present study.

## Introduction

Diabetes mellitus develops due to insufficient sensitivity and/or secretion of insulin.^1^ In particular, type 2 diabetes mellitus, which accounts for ∼90% of all patients with diabetes mellitus, mainly develops due to insufficient sensitivity to insulin. As a risk factor for insufficient sensitivity to insulin, the importance of obesity has been established.^2^ Obesity is also reported to be associated with health problems such as cancer and cardiovascular disease.^3^ The body mass index (BMI), waist circumference (WC), and WC/Height (Ht) ratio have been used as indices of obesity for a long time.^4,5^ However, these traditional anthropometric indices cannot distinguish between mass containing fat (fat mass) and fat-free mass, making accurate classification of risk for obesity-related complications difficult.^6,7^

To improve the limitations of traditional anthropometric indices, a body shape index (ABSI) and body roundness index (BRI) have been developed.^8,9^ The ABSI and BRI are reported to be comparable or superior predictive markers of mortality and metabolic syndrome than traditional anthropometric indices.^10–13^ These associations may be mediated by insulin resistance. A small number of studies to date have examined the associations between the ABSI and/or BRI and insulin sensitivity (SI) using fasting insulin.^14–17^ In adults without diabetes, the ABSI is weakly correlated with SI, whereas the BRI shows a stronger correlation with SI, comparable to traditional anthropometric indices.^14,15^ In obese and young persons, it is the BRI, not the ABSI that is reportedly correlated with SI.^16,17^ These epidemiological studies had small sample sizes and limitations in terms of age and glucose tolerance. To our knowledge, no study has examined the association between the ABSI and/or the BRI and indices of SI using the oral glucose tolerance test (OGTT) in a large and varied population.

The present study was performed to test the hypothesis that the ABSI and/or BRI is better related to SI than traditional anthropometric indices such as the BMI, WC, and WC/Ht. To test this hypothesis, the ABSI, BRI, and traditional anthropometric indices were compared with indices of SI using postload insulin levels based on a 75-g OGTT in a large sample divided mainly into three different groups: young persons with normal glucose tolerance (NGT), middle-aged persons with NGT, and middle-aged persons, including both those with NGT and those with glucose intolerance.

## Materials and Methods

### Participants

The study participants were 1270 medical students at Jichi Medical University, Tochigi, Japan (age <40 years) who had NGT, from among about 1400 students who had undergone a 75-g OGTT between December 2002 and April 2015. NGT was defined based on Japan Diabetes Society criteria [fasting plasma glucose (PG) <110 mg/dL and 120-min value <140 mg/Dl].^18^ The study was approved by the Ethics Committee at Jichi Medical University (approval no. EKI 09–45). Written, informed consent was obtained from all participants after providing full information on the purposes of the study (Jichi cohort). Data collection was consistent throughout the study period in the Jichi cohort.

Data from health examinees, 30–65 years of age, at Hokuriku Central Hospital, Toyama, Japan, were also analyzed. The detailed characteristics of the study population have been described elsewhere.^19,20^ Briefly, 2153 participants who visited the hospital between April 2006 and March 2010 were enrolled in this study after excluding those who had Hemoglobin A1c values ≥6.5%, who had a known history of diabetes mellitus and/or taking antidiabetic agents, who had undergone gastrectomy, who were taking steroids, or who were taking anticancer drugs. The study was approved by the Ethics Committee of the Hokuriku Central Hospital.^19,20^ Written, informed consent was obtained from all participants after providing full information on the purposes of the study (Hokuriku cohort).

### Measurements and calculation of SI

PG concentrations were measured using a glucose oxidase assay, and insulin levels were measured using an immunoradiometric assay for immunoreactive insulin (IRI) (Insulin RIA Beads II; Yamasa, Tokyo, Japan), as described previously (Jichi cohort).^21^ Serum IRI concentrations were determined using a chemiluminescence immunoassay (Siemens Healthcare Diagnostics, Tokyo, Japan) at a commercial laboratory (BML, Inc., Tokyo, Japan) (Hokuriku cohort).^19,20^ The antibodies used in both insulin assays did not cross-react with proinsulin. In the 75-g OGTT, PG and IRI levels were measured under fasting conditions (preloading) and 120 min after glucose loading; these are abbreviated as PG0 and PG120, and IRI0 and IRI120, respectively.

Similar to our previous studies,^21,22^ the following measures were used. Systemic SI as determined by the Matsuda index (ISI-Matsuda) was calculated as: ISI-Matsuda = 10,000/[sqrt (PG0 × PG120 × IRI0 × IRI120)].^23,24^ In addition, 1/homeostasis model assessment of insulin resistance (HOMA-IR) was used primarily as a measure of hepatic SI. HOMA-IR was calculated as [PG0 × IRI0/405].^25^ The units for PG and IRI were milligrams per deciliter and microunits per milliliter for calculating ISI-Matsuda and HOMA-IR. Negative values were treated as missing.

The 25th percentile for ISI-Matsuda and 1/HOMA-IR in the NGT of each cohort was adopted as the cutoff for decreased SI, that is, insulin resistance. The 25th percentile was adopted according to the previous study.^16^ In the Jichi cohort, an ISI-Matsuda of ≤6.1 and a 1/HOMA-IR of ≤0.562 were used. In the Hokuriku cohort, an ISI-Matsuda of ≤7.7 and a 1/HOMA-IR of ≤0.827 were used.

### Questionnaires and measurements of background factors

Data on age and sex were obtained through questionnaires. High-density lipoprotein cholesterol (HDL), triglyceride (TG), and total cholesterol (T-chol) levels were measured using serum collected under fasting conditions. The units for HDL, TG and T-Chol were milligrams per deciliter. The low-density lipoprotein cholesterol (LDL) concentration was calculated using the Friedewald formula (LDL = T-chol − HDL − TG/5).^26^ Blood pressure (BP) (systolic BP and diastolic BP) were measured after the participant had been seated at rest for 5 min.

### Measurements of anthropometric indices

BMI was calculated as the weight in kilograms divided by the height (Ht) in meters squared. WC was measured at the umbilical level with the subject standing.^27^ The WC/Ht ratio was also calculated. ABSI, which has been reported,^8^ was calculated as: WC/BMI^2/3^/Ht^1/2^. The BRI, which has also been reported,^9^ was calculated as: 364.2–365.5 × {1−[(1/2 × WC/π)/(0.5 × Ht)]^2^}^0.5^.

### Statistical analyses

JMP version 5.1 was used for all statistical analyses, except for the receiver-operating characteristic (ROC) curve analysis. Since almost none of the variables had a normal distribution, results are expressed as median (25th percentile, 75th percentile) values. The correlations of anthropometric indices with SI were tested using Spearman's rank-correlation coefficients on bivariate analysis.

ROC curves and the area under the ROC curves (AUCs) were used to assess the ability of each anthropometric index to detect insulin resistance, using EZR ver. 1.61 (Saitama Medical Center, Jichi Medical University, Saitama, Japan).^28^ If the lower limit of the 95% confidence interval for the AUC of an anthropometric index was below 0.50, that index was considered to not have the ability to detect insulin resistance. Differences in two AUCs were assessed by the method described by Delong.^29^ Optimal cutoff values of anthropometric indices were determined by maximization of the Youden index (sensitivity+specificity −1). For all statistical tests, values of *P* < 0.05 were considered significant.

## Results

### Characteristics of the entire cohort

The characteristics of the study participants by sex are shown in Table 1. The Jichi cohort (*n* = 1270) included only persons with NGT and was a young cohort with few cases of obesity, hypertension, and dyslipidemia. The participants in the Hokuriku cohort were sorted into an NGT-only group (*n* = 1531) and a group that included those with NGT and those with glucose intolerance (*n* = 2153). Both groups in the Hokuriku cohort consisted of middle-aged persons who had higher BMI, WC, WC/Ht ratio, BP, lipids, and glucose than the young persons with NGT (the Jichi cohort). The Hokuriku cohort included 622 persons with glucose intolerance (nondiabetic hyperglycemia, *n* = 547; newly diagnosed diabetes mellitus, *n* = 75), accounting for 29% of the total cohort. The group in the Hokuriku cohort that included those with NGT and those with glucose intolerance did not appear to have any major differences in age, BMI, WC, height, WC/Ht ratio, BP, or lipids compared with the NGT group of the same cohort; however, their glucose levels were higher 120 min after glucose loading, and their ISI-Matsuda was low.

**Table 1.** Characteristics of the Participants in the Jichi and Hokuriku Cohorts

	Jichi cohort (young NGT)		Hokuriku cohort (middle-aged NGT)		Hokuriku cohort, including NGT and glucose intolerance (middle-aged overall)	
	Men (*n* = 979)	Women (*n* = 291)	Men (*n* = 1019)	Women (*n* = 512)	Men (*n* = 1468)	Women (*n* = 685)
Age (years)	23 (22, 23)	23 (22, 24)	52 (46, 58)	55 (50, 59)	53 (47, 59)	56 (50, 59)
BMI (kg/m^2^)	21.8 (20.5, 23.3)	20.0 (18.8, 21.1)	23.9 (22.2, 25.6)	22.3 (20.6, 24.6)	24.1 (22.3, 25.9)	22.5 (20.7, 24.6)
WC (cm)	77 (73, 81)	68 (64, 72)	84 (79, 89)	80 (74, 87)	85 (80, 90)	80 (74, 87)
Height (cm)	172 (169, 176)	160 (156, 163)	170 (166, 174)	156 (153, 160)	170 (166, 174)	157 (153, 160)
WC/Ht ratio	0.44 (0.42, 0.47)	0.43 (0.41, 0.45)	0.49 (0.47, 0.52)	0.51 (0.47, 0.55)	0.50 (0.47, 0.53)	0.52 (0.47, 0.55)
ABSI	0.75 (0.73, 0.77)	0.74 (0.70, 0.77)	0.77 (0.75, 0.80)	0.80 (0.77, 0.84)	0.78 (0.76, 0.80)	0.80 (0.77, 0.84)
BRI	2.38 (2.02, 2.82)	2.12 (1.77, 2.50)	3.25 (2.77, 3.79)	3.60 (2.81, 4.26)	3.33 (2.85, 3.91)	3.65 (2.86, 4.44)
SBP (mmHg)	120 (115, 128)	108 (103, 115)	129 (117, 142)	122 (111, 138)	131 (119, 144)	122 (111, 138)
DBP (mmHg)	67 (63, 73)	63 (60, 67)	80 (74, 88)	75 (68, 83)	82 (75, 90)	75 (68, 83)
HDL (mg/dL)	59 (52, 67)	68 (59, 77)	55 (47, 64)	64 (56, 76)	55 (47, 64)	64 (56, 75)
TG (mg/dL)	63 (49, 86)	52 (40, 67)	113 (82, 155)	82 (64, 114)	117 (86, 163)	85 (64, 121)
LDL (mg/dL)	90 (76, 105)	96 (81, 114)	125 (106, 145)	129 (108, 150)	126 (107, 147)	132 (111, 153)
PG0 (mg/dL)	88 (82, 94)	85 (80, 91)	96 (92, 101)	93 (89, 97)	99 (93, 105)	95 (90, 101)
PG120 (mg/dL)	90 (78, 102)	94 (80, 107)	108 (94, 121)	105 (93, 118)	116 (99, 138)	112 (98, 135)
IRI0 (μU/mL)	5.8 (4.3, 8.1)	6.1 (4.6. 8.5)	3.8 (2.8, 5.2)	3.6 (2.9, 4.9)	3.9 (2.8, 5.4)	3.7 (2.9, 5.1)
IRI120 (μU/mL)	23.2 (13.9, 37.6)	39.0 (25.5, 58.2)	21.6 (13.9, 33.7)	21.5 (15.4, 32.3)	24.0 (15.4, 39.6)	24.5 (16.6, 38.1)
HOMA-IR	1.26 (0.90, 1.77)	1.30 (0.94, 1.80)	0.89 (0.65, 1.23)	0.85 (0.65, 1.13)	0.95 (0.69, 1.33)	0.88 (0.66, 1.25)
1/HOMA-IR	0.8 (0.6, 1.1)	0.8 (0.6, 1.1)	1.1 (0.8, 1.5)	1.2 (0.9, 1.5)	1.1 (0.7, 1.5)	1.1 (0.8, 1.5)
ISI-Matsuda	10.1 (6.4, 14.6)	7.4 (5.3, 10.0)	11.2 (7.5, 16.4)	11.5 (8.1, 16.3)	9.9 (6.3, 14.6)	10.1 (6.7, 14.7)
NDH/Diabetes (number)	—	—	—	—	395/54	152/21

Date are shown as median (25th percentile, 75th percentile) values.

ABSI, a body shape index; BMI, body mass index; BRI, body roundness index; DBP, diastolic blood pressure; Ht, height; HDL, high-density lipoprotein cholesterol; HOMA-IR, homeostasis model assessment of insulin resistance; IRI, immunoreactive insulin; ISI-Matsuda, Matsuda index; LDL, low-density lipoprotein cholesterol; PG, plasma glucose; NGT, normal glucose tolerance; SBP, systolic blood pressure; NDH, nondiabetic hyperglycemia; TG, triglyceride; WC, waist circumference.

The ABSI did not differ between any of the cohorts or between the sexes, ranging from about 0.7 to 0.8.

The BRI was higher in the Hokuriku cohort than in the Jichi cohort. Men had a higher BRI and BMI than women in the Jichi cohort. The BMI was lower in women in the Hokuriku cohort, but the women had a higher BRI than the men.

### Relationships of anthropometric indices with SI in each cohort

In both men and women in the Jichi cohort (young persons with NGT), BRI showed similar or high inverse correlations with 1/HOMA-IR and ISI-Matsuda compared with the BMI, WC, and WC/Ht ratio, whereas the ABSI showed a weak inverse correlation (Table 2). In both men and women among the middle-aged persons with NGT and glucose intolerance, those with NGT, and those with glucose intolerance in the Hokuriku cohort, the BRI showed almost the same inverse correlation with 1/HOMA-IR and ISI-Matsuda compared with the BMI, WC, and WC/Ht ratio, whereas the ABSI showed a weak inverse correlation (Table 2). Spearman's ρ values for SI of the BRI were higher in men in the Jichi cohort, and similarly, those for SI of the BRI were higher in men with NGT and glucose intolerance, and in men with glucose intolerance in the Hokuriku cohort. Overall, Spearman's ρ values for each anthropometric index and SI for both men and women were higher in the Hokuriku cohort than in the Jichi cohort (Table 2).

**Table 2.** Nonparametric Spearman's Rank Coefficients of Anthropometric Indices for 1/HOMA-IR and ISI-Matsuda by Sex

	For 1/HOMA-IR				For ISI-Matsuda			
	Men		Women		Men		Women	
	ρ	P	ρ	P	ρ	P	ρ	P
Jichi cohort (young NGT)								
BMI	−0.23	<0.0001	−0.18	<0.01	−0.16	<0.0001	−0.16	<0.01
WC	−0.27	<0.0001	−0.18	<0.01	−0.23	<0.0001	−0.21	<0.001
WC/Ht ratio	−0.27	<0.0001	−0.17	<0.01	−0.27	<0.0001	−0.21	<0.001
ABSI	−0.15	<0.0001	−0.047	0.43	−0.22	<0.0001	−0.091	0.12
BRI	−0.27	<0.0001	−0.17	<0.01	−0.27	<0.0001	−0.21	<0.001
Hokuriku cohort, including NGT and glucose intolerance (middle-aged overall)								
BMI	−0.67	<0.0001	−0.51	<0.0001	−0.83	<0.0001	−0.43	<0.0001
WC	−0.73	<0.0001	−0.46	<0.0001	−0.91	<0.0001	−0.40	<0.0001
WC/Ht ratio	−0.78	<0.0001	−0.46	<0.0001	−1.00	<0.0001	−0.42	<0.0001
ABSI	−0.31	<0.0001	−0.12	<0.01	−0.42	<0.0001	−0.10	<0.01
BRI	−0.78	<0.0001	−0.46	<0.0001	−1.00	<0.0001	−0.42	<0.0001
Hokuriku cohort (middle-aged NGT)								
BMI	−0.50	<0.0001	−0.46	<0.0001	−0.44	<0.0001	−0.42	<0.0001
WC	−0.51	<0.0001	−0.42	<0.0001	−0.45	<0.0001	−0.39	<0.0001
WC/Ht ratio	−0.49	<0.0001	−0.42	<0.0001	−0.46	<0.0001	−0.41	<0.0001
ABSI	−0.12	<0.001	−0.11	<0.05	−0.14	<0.0001	−0.11	<0.05
BRI	−0.49	<0.0001	−0.42	<0.0001	−0.46	<0.0001	−0.41	<0.0001
Hokuriku cohort (middle-aged glucose intolerance)								
BMI	−0.68	<0.0001	−0.58	<0.0001	−0.84	<0.0001	−0.43	<0.0001
WC	−0.74	<0.0001	−0.49	<0.0001	−0.92	<0.0001	−0.38	<0.0001
WC/Ht ratio	−0.77	<0.0001	−0.50	<0.0001	−1.00	<0.0001	−0.38	<0.0001
ABSI	−0.28	<0.0001	−0.12	0.12	−0.39	<0.0001	−0.072	0.35
BRI	−0.77	<0.0001	−0.50	<0.0001	−1.00	<0.0001	−0.38	<0.0001

**FIG. 1.**
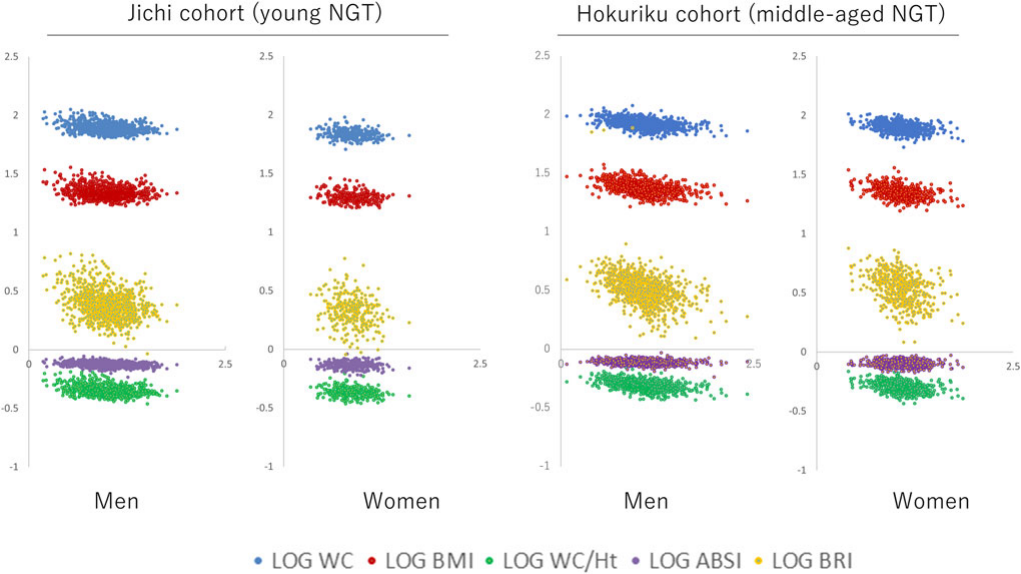
Relationships between the common LOGs of anthropometric indices (*vertical axis*) and LOG ISI-Matsuda (*horizontal axis*). LOG WC: blue points; LOG BMI: red points; LOG WC/Ht: green points; LOG ABSI: purple points; LOG BRI: yellow points. ABSI, a body shape index; BMI, body mass index; BRI, body roundness index; Ht, height; LOG, logarithm; NGT, normal glucose tolerance; WC, waist circumference.

The distribution of each of the anthropometric indices for ISI-Matsuda in participants with NGT in both cohorts is shown in Fig. 1. The range of ISI-Matsuda and each of the anthropometric indices increased more in middle-aged persons with NGT in the Hokuriku cohort than in the Jichi cohort (young persons with NGT). ISI-Matsuda showed a particularly wide distribution (Table 1 and Fig. 1).

### ROC analyses

The results of the ROC analyses using each of the anthropometric indices for the presence or absence of insulin resistance based on ISI-Matsuda are shown in Table 3. The active model of the ROC curve could not be generated by EZR for women in the Jichi cohort. The AUCs were significant for the ability of the BMI, WC, WC/Ht ratio, ABSI, and BRI in men of the three cohorts and women of the Hokuriku cohort to identify insulin resistance. The AUCs were not significant for the ability of the ABSI to identify insulin resistance in women of the Jichi cohort. In addition, the ABSI showed the lowest AUCs in all men and women of these cohorts.

**Table 3.** Area Under the Curve, Sensitivity and Specificity of Cutoff Values of Anthropometric Indices for the Presence of Insulin Resistance by ISI-Matsuda in Both Sexes

	For the presence of insulin resistance by ISI-Matsuda							
	Men				Women			
	AUC (95% CI)	Cut-off value	Sensitivity (%)	Specificity (%)	AUC (95% CI)	Cutoff value	Sensitivity (%)	Specificity (%)
Jichi cohort (young NGT)								
BMI	0.63 (0.59–0.68)	22.8	50	72	0.57 (0.50–0.64)	21.9	25	91
WC	0.68 (0.64–0.72)^a^	78.0	66	62				
WC/Ht ratio	0.69 (0.64–0.73)^b^	0.45	68	64	0.61 (0.54–0.68)	0.44	56	64
ABSI	0.65 (0.61–0.69)	0.76	53	70	0.56 (0.49–0.62)	0.76	46	66
BRI	0.69 (0.64–0.73)^b^	2.48	68	64	0.61 (0.54–0.68)	2.22	56	64
Hokuriku cohort, including NGT and glucose intolerance (middle-aged overall)								
BMI	0.91 (0.90–0.93)^d^	25.0	79	86	0.73 (0.69–0.77)^c^	23.3	64	71
WC	0.95 (0.94–0.96)^e^	87.0	87	87	0.72 (0.68–0.76)^c^	83.0	65	69
WC/Ht ratio	1.00 (1.00–1.00)^c^	0.51	100	100	0.72 (0.68–0.76)^c^	0.53	68	68
ABSI	0.69 (0.67–0.72)	0.78	62	66	0.56 (0.52–0.61)	0.80	53	58
BRI	1.00 (1.00–1.00)^c^	3.63	100	100	0.72 (0.68–0.76)^c^	3.84	68	68
Hokuriku cohort (middle-aged NGT)								
BMI	0.76 (0.72–0.79)^c^	23.8	81	58	0.74 (0.69–0.79)^c^	23.2	67	71
WC	0.76 (0.72–0.79)^c^	85.0	75	64	0.73 (0.68–0.78)^c^	81.0	74	62
WC/Ht ratio	0.77 (0.73–0.80)^c^	0.49	88	52	0.75 (0.70–0.79)^c^	0.52	72	69
ABSI	0.58 (0.54–0.62)	0.77	64	50	0.58 (0.52–0.63)	0.81	53	59
BRI	0.77 (0.73–0.80)^c^	3.08	88	52	0.75 (0.70–0.79)^c^	3.83	72	69
Hokuriku cohort (middle-aged glucose intolerance)								
BMI	0.91 (0.88–0.93)^f^	25.0	81	84	0.69 (0.61–0.77)^g^	23.3	63	68
WC	0.95 (0.94–0.97)^e^	88.0	87	92	0.69 (0.60–0.77)^h^	80.0	75	60
WC/Ht ratio	1.00 (1.00–1.00)^c^	0.51	100	100	0.68 (0.59–0.76)^i^	0.51	75	58
ABSI	0.69 (0.64–0.74)	0.77	76	53	0.55 (0.45–0.65)	0.78	74	42
BRI	1.00 (1.00–1.00)^c^	3.63	100	100	0.68 (0.59–0.76)^i^	3.50	75	58

In female of Jichi cohort, the active model of the ROC curve for WC could not be generated by EZR.

^a^ Versus BMI, *P* < 0.001.

^b^ Versus BMI, *P* < 0.0001.

^c^ Versus ABSI, *P* < 0.0001.

^d^ Versus WC, WC/Ht ratio, ABSI and BRI, *P* < 0.0001.

^e^ Versus WC/Ht ratio, ABSI and BRI, *P* < 0.0001.

^f^ Versus WC, *P* < 0.001/Versus WC/Ht ratio, ABSI and BRI, *P* < 0.0001.

^g^ Versus ABSI, *P* < 0.05.

^h^ Versus ABSI, *P* < 0.001.

^i^ Versus ABSI, *P* < 0.01.

AUC, area under the receiver-operating characteristic curve; CI, confidence interval; ROC, receiver-operating characteristic.

In both men and women of these cohorts, the BRI and WC/Ht ratio generally scored the highest and showed almost the same AUCs. In women in the Hokuriku cohort, the BMI showed a similar AUC to the BRI and WC/Ht ratio. The AUCs for the BRI and WC/Ht ratio were the highest in middle-aged men, including those with NGT and glucose intolerance, and those with glucose intolerance (Hokuriku cohort).

The results of ROC analyses using each of the anthropometric indices for the presence or absence of insulin resistance based on 1/HOMA-IR were mostly similar to those based on ISI-Matsuda (Supplementary Table S1).

## Discussion

The present study investigated whether the BRI and ABSI correlate better with SI than traditional anthropometric indices such as the BMI, WC, and WC/Ht ratio. The BRI showed comparable correlations to the BMI, WC, and WC/Ht ratio in both men and women among young persons, middle-aged persons with NGT, and middle-aged persons, including those with NGT and those with glucose intolerance. However, in the participants of the present study, the BRI did not correlate better with SI than traditional anthropometric indices, especially the WC/Ht ratio. On the other hand, the correlation between the ABSI and SI was weaker than for the other anthropometric indices in all groups. The correlation coefficient between each anthropometric index and SI was higher in middle-aged persons than in young persons. On ROC curve analysis for detection of insulin resistance, the ABSI showed a smaller AUC than the other anthropometric indices, and the BRI and WC/Ht ratio showed similar AUCs in both men and women. The largest AUC was seen in middle-aged men, including those with glucose intolerance.

So far, the BRI has been reported to show similar or stronger correlations with SI and cardiovascular and metabolic risks, such as metabolic syndrome, than the conventional BMI, WC, and ABSI in populations with limited ranges in terms of age, glucose tolerance, and other factors.^12–17^ The results of the present study are consistent with results to date. In epidemiological studies, carrying out an OGTT is difficult, and postload insulin levels in particular are often impossible to measure. Many studies therefore cannot measure SI such as by ISI-Matsuda. In the present study, ISI-Matsuda was used as an index of SI, and the correlation with each of the anthropometric indices could be calculated in a population of varied age and glucose tolerance.

The BRI was higher in the middle-aged Hokuriku cohort than in the young Jichi cohort in both men and women. The BRI was higher in young men than in young women, and higher in middle-aged women than middle-aged men. This is thought to be because, in the Jichi cohort, the WC of women was much smaller than that of men, but the sex difference in Ht was small, whereas in the Hokuriku cohort, women were shorter in Ht, but had a larger WC. Compared with the ABSI, the BRI showed a better correlation with SI in both cohorts. The BRI is more reflective of central obesity and is thought to show a good correlation with SI.^9,30^

On the other hand, the ABSI values were similar in all groups of both cohorts and correlated poorly with SI. The methods of calculating the BRI and ABSI differ, in that the WC is divided by BMI^2/3^ in the latter, suggesting a possible weakening of the correlation of WC to SI. In the present study, the ABSI correlated poorly with the other anthropometric indices in men and women of all cohorts, and, in particular, the correlation between the ABSI and BMI was not significant (Spearman's rank correlation, data not shown). This also suggests that the ABSI has different properties from other body anthropometric indices.

SI and each of the anthropometric indices showed a better correlation in the middle-aged Hokuriku cohort than in the young Jichi cohort. In both men and women of the middle-aged Hokuriku cohort, BMI and WC increased, with a high possibility of many participants with low physical activity. On the other hand, in the Jichi cohort, the physical activity of individuals could not be analyzed, but ∼80% of the participants exercised regularly. The differences in physical activity likely affected the correlation between SI and each anthropometric index. Furthermore, in the middle-aged Hokuriku cohort, the increased ranges of SI and each anthropometric index may have been another factor for the good correlation.

On ROC examination for the presence or absence of insulin resistance based on ISI-Matsuda, the AUC for the ABSI was small in all cohorts. This is consistent with the poor correlation between SI and the ABSI. The BRI is calculated using WC and Ht, which affected the finding that the AUCs of the BRI and WC/Ht ratio were similar. In fact, the correlations between the anthropometric indices in the present study were very strong for the BRI and WC/Ht ratio in men and women in all cohorts (Spearman's ρ values were nearly 1). In an earlier report, the AUCs of the BRI and WC/Ht ratio for insulin resistance were similar,^15^ as in the present study. The AUC was generally larger in the middle-aged Hokuriku cohort compared with the young Jichi cohort. In the middle-aged Hokuriku cohort, the BRI and WC/Ht ratio in men and the BMI, BRI, and WC/Ht ratio in women showed high sensitivity and specificity and a large AUC, whereas the BRI and WC/Ht ratio showed the highest sensitivity and specificity and the largest AUC, particularly in men, including those with glucose intolerance.

The results of these ROC analyses were consistent with the correlations of anthropometric indices to the SI. The ability of the BRI to detect insulin resistance was comparable to that of traditional anthropometric markers such as the WC/Ht ratio.

Numerous reports have examined the correlations between SI and anthropometric indices such as the BMI,^14–17,31–34^ and many studies included a large number of obese persons. In the present study, a linear correlation was seen between anthropometric indices and SI in two cohorts containing mostly nonobese persons (Fig. 1). East Asians are known to have a lower BMI, but have a higher likelihood of developing diabetes mellitus than Westerners.^35,36^ In young and middle-aged East Asians, maintaining an appropriate body weight may help maintain SI and inhibit the development of glucose intolerance.

Unexpectedly, indices of SI tended to be better in the middle-aged Hokuriku cohort than in the young Jichi cohort, despite increases in the BMI and WC. One simple explanation for this is the difference in the method of measuring insulin. However, some young persons are known to excrete too much insulin to glucose load; such hypersecretion of insulin may be a precursor to glucose intolerance.^37,38^ Many reports have noted that insulin secretion decreases with aging,^39,40^ and differences in insulin levels may be due to differences in age. The above points meant that comparison of insulin levels and indices of SI between the two cohorts was difficult, and a combined analysis of both groups could not be performed. However, the aim of the present study was not to compare the indices of SI of the two cohorts. The investigation of the correlation between anthropometric indices and SI was performed in each cohort, and cutoff values were set in each cohort in the ROC analysis for detection of insulin resistance. Therefore, differences in insulin-measuring methods did not appear to affect the results.

Further limitations of the present study were that no elderly persons or a large number of diabetic patients could be examined, few obese persons were included, and hip circumference (Hc) could not be measured. Unfortunately, the relative merits of the WC/Ht ratio and the WC/Hc ratio could not be investigated. In addition, the participants of the present study were limited to Japanese people. The anthropometric indices and SI of Japanese people and Westerners differ greatly, and HOMA-IR is reported to differ even with an equivalent BMI.^41^ It is unclear whether the results also apply to other populations, such as Westerners. The use of insulin sensitizers as antidiabetic agents may decrease insulin levels, and thereby, affect SI indices. However, participants taking antidiabetic agents were excluded in the Hokuriku cohort, and not included in the Jichi cohort. The use of antihypertensive agents may also increase and/or decrease SI. In the overall Hokuriku cohort, 222 men and 83 women had taken antihypertensive agents.

Spearman's rank coefficients of anthropometric indices for 1/HOMA-IR and ISI-Matsuda, excluding those taking antihypertensive agents were exactly identical to those of the overall Hokuriku cohort (data not shown). Effects of the use of antihypertensive agents seem to be minor.

## Conclusions

In conclusion, in young and middle-aged Japanese people, the BRI, and not the ABSI, was better correlated with the SI, and the BRI was comparable to the WC/Ht ratio in detecting insulin resistance. In addition to traditional anthropometric indices, such as the BMI, the BRI may be clinically useful as a marker for predicting the onset of metabolic syndrome and diabetes mellitus. A follow-up study of the development of glucose intolerance in the Jichi and Hokuriku cohorts is in progress.

## Supplementary Material

Supplemental data

## Data Availability

The datasets generated and/or analyzed during the present study are not publicly available, but they are available from the corresponding author on reasonable request.
