# Supplementary material for: Body Roundness Index Is Better Correlated with Insulin Sensitivity than Body Shape Index in Young and Middle-Aged Japanese Persons
Source: Metab Syndr Relat Disord. 2024 Mar 14;22(2):151–9. doi: 10.1089/met.2023.0175 (PMC10951619; doi:10.1089/met.2023.0175)
Supplement: Supplemental data [file Suppl_TableS1.docx]

Supplementary Table. Area under the curve, sensitivity and specificity of cut-off values of anthropometric indices for the presence of insulin resistance by 1/HOMA-IR in both sexes

|  | For the presence of insulin resistance by 1/HOMA-IR | | | | | | | |
| --- | --- | --- | --- | --- | --- | --- | --- | --- |
|  | Men | | | | Women | | | |
|  | AUC (95%CI) | Cut-off value | Sensitivity (%) | Specificity (%) | AUC (95%CI) | Cut-off value | Sensitivity (%) | Specificity (%) |
| Jichi cohort  (young NGT) |  |  |  |  |  |  |  |  |
| BMI | 0.64 (0.59-0.68) ^a^ | 21.8 | 66 | 55 | 0.62 (0.54-0.70) ^c^ | 21.0 | 45 | 78 |
| WC | 0.66 (0.62-0.70) ^b^ | 78.0 | 64 | 63 |  |  |  |  |
| WC/Ht ratio | 0.65 (0.61-0.69) ^b^ | 0.45 | 61 | 63 | 0.60 (0.53-0.68) | 0.42 | 77 | 41 |
| ABSI | 0.58 (0.54-0.62) | 0.75 | 56 | 59 | 0.49 (0.41-0.56) | 0.72 | 45 | 60 |
| BRI | 0.65 (0.61-0.69) ^b^ | 2.48 | 61 | 63 | 0.60 (0.53-0.68) | 1.91 | 77 | 41 |
| Hokuriku cohort including NGT and glucose intolerance  (middle-aged overall) |  |  |  |  |  |  |  |  |
| BMI | 0.85 (0.83-0.87) ^e^ | 24.6 | 78 | 75 | 0.78 (0.74-0.82) ^d^ | 23.1 | 73 | 71 |
| WC | 0.88 (0.86-0.90) ^f^ | 87.0 | 79 | 80 | 0.76 (0.73-0.80) ^d^ | 83.0 | 73 | 69 |
| WC/Ht ratio | 0.91 (0.90-0.93) ^d^ | 0.51 | 82 | 83 | 0.76 (0.72-0.80) ^d^ | 0.51 | 67 | 74 |
| ABSI | 0.66 (0.63-0.69) | 0.78 | 61 | 64 | 0.57 (0.52-0.62) | 0.80 | 55 | 58 |
| BRI | 0.91 (0.90-0.93) ^d^ | 3.59 | 82 | 83 | 0.76 (0.72-0.80) ^d^ | 4.01 | 67 | 74 |
| Hokuriku cohort  (middle-aged NGT) |  |  |  |  |  |  |  |  |
| BMI | 0.77 (0.74-0.80) ^d^ | 24.3 | 73 | 67 | 0.75 (0.70-0.80) ^d^ | 23.3 | 66 | 72 |
| WC | 0.77 (0.74-0.80) ^d^ | 85.0 | 76 | 65 | 0.74 (0.70-0.79) ^d^ | 83.0 | 69 | 70 |
| WC/Ht ratio | 0.76 (0.73-0.80) ^d^ | 0.48 | 91 | 49 | 0.74 (0.69-0.79) ^d^ | 0.53 | 67 | 72 |
| ABSI | 0.55 (0.52-0.59) | 0.77 | 61 | 47 | 0.57 (0.51-0.62) | 0.81 | 60 | 50 |
| BRI | 0.76 (0.73-0.80) ^d^ | 3.03 | 91 | 49 | 0.74 (0.69-0.79) ^d^ | 3.92 | 67 | 72 |
| Hokuriku cohort  (middle-aged glucose intolerance) |  |  |  |  |  |  |  |  |
| BMI | 0.84 (0.81-0.88) ^e^ | 25.7 | 76 | 77 | 0.81 (0.74-0.87) ^d^ | 23.3 | 82 | 68 |
| WC | 0.89 (0.86-0.92) ^g^ | 87.0 | 77 | 84 | 0.78 (0.71-0.85) ^d^ | 83.0 | 79 | 65 |
| WC/Ht ratio | 0.91 (0.89-0.94) ^d^ | 0.54 | 77 | 99 | 0.78 (0.71-0.85) ^d^ | 0.55 | 61 | 81 |
| ABSI | 0.67 (0.62-0.72) | 0.78 | 70 | 59 | 0.58 (0.50-0.67) | 0.81 | 62 | 58 |
| BRI | 0.91 (0.89-0.94) ^d^ | 4.12 | 77 | 99 | 0.78 (0.71-0.85) ^d^ | 4.45 | 61 | 81 |

Abbreviations: HOMA-IR, homeostasis model assessment of insulin resistance; NGT, normal glucose tolerance; AUC, area under the receiver operating characteristic curve; CI, confidence interval; BMI, body mass index; WC, waist circumference; Ht, height; ABSI, a body shape index; BRI, body roundness index.

In female of Jichi cohort, the active model of the receiver-operating characteristic curve for WC could not be generated by EZR.

^a^ vs ABSI, *P*<0.05

^b^ vs ABSI, *P*<0.001

^c^ vs ABSI, *P*<0.01

^d^ vs ABSI, *P*<0.0001

^e^ vs WC, *P*<0.001 / vs WC/Ht ratio, ABSI and BRI, *P*<0.0001

^f^ vs WC/Ht ratio, ABSI and BRI, *P*<0.0001

^g^ vs WC/Ht ratio and BRI, *P*<0.05 / vs ABSI, *P*<0.0001
